# Identification of tumor tissue-derived DNA methylation biomarkers for the detection and therapy response evaluation of metastatic castration resistant prostate cancer in liquid biopsies

**DOI:** 10.1186/s12943-021-01445-0

**Published:** 2022-01-03

**Authors:** Thomas Dillinger, Raheleh Sheibani-Tezerji, Walter Pulverer, Ines Stelzer, Melanie R. Hassler, Janine Scheibelreiter, Carlos Uziel Pérez Malla, Madeleine Kuroll, Sandra Domazet, Elisa Redl, Sarah Ely, Stefanie Brezina, Andreas Tiefenbacher, Katharina Rebhan, Nicolai Hübner, Bernhard Grubmüller, Markus Mitterhauser, Marcus Hacker, Andreas Weinhaeusel, Judit Simon, Markus Zeitlinger, Andrea Gsur, Gero Kramer, Shahrokh F. Shariat, Lukas Kenner, Gerda Egger

**Affiliations:** 1 Ludwig Boltzmann Institute Applied Diagnostics, Vienna, Austria; 2 Department of Pathology, Medical University of Vienna, Vienna, Austria; 3 Health & Environment Department, Molecular Diagnostics, AIT-Austrian Institute of Technology GmbH, Vienna, Austria; 4 Department of Health Economics, Center for Public Health, Medical University of Vienna, Vienna, Austria; 5 Department of Urology, Medical University Vienna, Vienna, Austria; 6 Department of Clinical Pharmacology, Medical University of Vienna, Vienna, Austria; 7 Institute of Cancer Research, Department of Medicine I, Medical University of Vienna, Vienna, Austria; 8 Division of Nuclear Medicine, Department of Biomedical Imaging and Image-Guided Therapy, Vienna, Austria; 9 Comprehensive Cancer Center, Medical University of Vienna, Vienna, Austria; 10 Department of Urology, University of Texas Southwestern Medical Center, Dallas, TX USA; 11 Department of Urology, Weill Cornell Medical College, New York, NY USA; 12 Department of Urology, Second Faculty of Medicine, Charles University, Prague, Czech Republic; 13 Karl Landsteiner Institute of Urology and Andrology, Vienna, Austria; 14 Division of Urology, Department of Special Surgery, Jordan University Hospital, The University of Jordan, Amman, Jordan; 15 European Association of Urology Research Foundation, Arnhem, The Netherlands; 16 Unit of Laboratory Animal Pathology, University of Veterinary Medicine, Vienna, Austria; 17 Christian Doppler Laboratory for Applied Metabolomics, Medical University of Vienna, Vienna, Austria

## Main text

Prostate cancer (PCa) is among the most common cancers in men worldwide [1] and comprises a highly heterogenous disease, which ranges from indolent localized cancer to aggressive high-risk stages, including metastatic hormone sensitive or hormone refractory PCa. Due to the limited specificity and sensitivity of current biomarkers such as PSA [2], there is an urgent need for better biomarkers that can reliably differentiate benign from malignant prostate conditions, localized from metastatic, as well as aggressive from indolent disease. Furthermore, the development of predictive biomarkers that allow for better patient stratification and of biomarkers for early monitoring of treatment response is of utmost importance [3].

Treatment options for PCa are mostly based on non-targeted therapies and include radical prostatectomy, hormonal treatment using androgen deprivation therapy (ADT), androgen receptor (AR) signaling-targeting agents, chemotherapy or radiation, depending on disease state and risk classification [4, 5]. Despite good initial response to ADT, tumors eventually progress to metastatic castration resistant PCa (mCRPC). In this setting, recent treatments including poly(ADP-ribose) polymerase inhibitors (PARPi) for tumors harboring mutations in DNA repair genes, or radionuclide therapy using ^177^Lu-PSMA for PSMA positive cancers have shown promising results [5].

Liquid biopsy assays, analyzing circulating free tumor DNA (ctDNA) or circulating tumor cells (CTCs) in plasma or other body fluids have proven as a useful source for biomarkers and have already entered the clinics for companion diagnostic use [6]. Aside from genetic alterations, epigenetic tumor-specific changes including DNA methylation are measurable in ctDNA and CTCs and their potential as diagnostic, prognostic and predictive epigenetic biomarkers has been demonstrated in a large number of studies although only few have made it into clinical practice, yet [7].

In this study, we investigated the suitability of DNA methylation-based biomarkers for non-invasive PCa diagnostics. Based on experiments and *in silico* analyses we identified two DNA methylation signatures, which could be used as minimal-invasive markers in liquid biopsies for the detection of methylated ctDNA. These signatures allowed for the classification of mCRPC with high specificity and sensitivity and were able to distinguish responders from non-responders following different treatment modalities. Importantly, several individual marker genes had prognostic potential for radiographic progression free survival independent of other clinical variables.

## Results and discussion

### Identification of tissue-specific DNA methylation markers

**Fig. 1:**
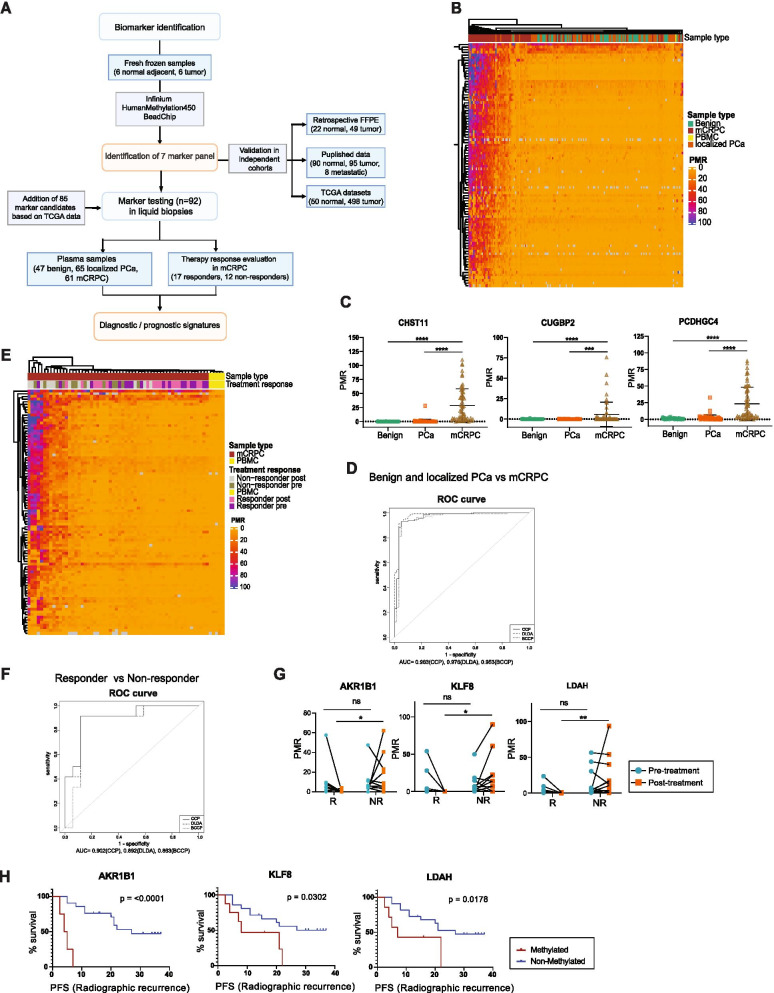
Identification and liquid biopsy testing of DNA methylation-based markers. **A** Experimental Workflow. Flow chart indicates biomarker identification, validation and analysis in different patient cohorts and publicly available datasets. **B** Unsupervised clustering of PMR-values resulting from MSRE-qPCR analysis of ccfDNA isolated from plasma of indicated patient groups for 92 marker candidates (*n*=47 benign, *n*=65 localized PCa, *n*=61 mCRPC, *n*=1 PBMC). **C** DNA methylation levels of ccfDNA isolated from plasma in patients with localized PCa or mCRPC versus controls for three signature genes. Differences between the three groups were assessed using one-way ANOVA (*CHST11*, *PCDHGC4 n*=47 benign, *n*=65 localized PCa, *n*=61 mCRPC, *CUGBP2 n*=46 benign, n=62 localized PCa, n=54 mCRPC; **** *p* < 0.0001). **D** ROC-curve analysis based on the three gene signature as in (**C**) for classification of mCRPC samples compared to benign and localized PCa patients combined, calculated with different prediction algorithms, ((Bayesian) Compound Covariate Predictor (BCCP, CCP) and Diagonal Linear Discriminant Analysis (DLDA)) using recursive feature elimination (*n*=112 benign + localized PCa, *n*=61 mCRPC). **E** Unsupervised clustering of PMR-values resulting from MSRE-qPCR of ccfDNA isolated from plasma of mCRPC patients before and after treatment, or healthy PBMC controls for 92 marker candidates (*n*=17 responders, *n*=12 non-responders, *n*=5 PBMC). **F** ROC-curve analysis based on a 3-gene signature (*AKR1B1*, *KLF8, LDAH*) for classification of responders versus non-responders. Calculations were performed using BCCP, CCP and DLDA with recursive feature elimination using BRB array tools software. **G** Methylation levels of the three signature genes for individual responders and non-responders pre- and post-treatment (*n*=17 responder, *n*=12 non-responder ** *p* < 0.01, * *p* < 0.05, ns *p* > 0.05; two-way ANOVA). **H** Kaplan-Meier-Analysis for radiographic progression-free survival (rPFS) of three signature genes using post-treatment samples (*LDAH, KLF8 n*=17 responder, *n*=12 non-responder; *AKR1B1 n*=15 responder, *n*=10 non-responder; *p* values shown on each plot calculated with Mantel-Cox-test, censored subjects indicated on plots by strokes)

Differences in DNA methylation profiles between normal and PCa tissue were shown in several studies to reveal potential DNA methylation-based biomarkers for PCa detection [8]. In order to define suitable epigenetic markers for non-invasive diagnostics, we combined experimental and *in silico* data to derive 92 methylation markers that were significantly differentially methylated between tumor and normal adjacent tissues, which we subsequently tested on ctDNA of PCa patients (Fig. 1A).

First, we used the Infinium HumanMethylation450 BeadChip array and probed DNA isolated from six localized PCa tissues compared to six adjacent normal tissues (Table S1). From these analyses, 7 genes including *SERPINB1*, *ACSS3*, *SCGB3A1, NKX2-6, HOXA7, CRABP2* and *DHRS4L2* were found significantly hypermethylated in the tumor samples compared to normal adjacent tissues (Fig. S1A). Methylation levels of those genes continually rose from benign to PCa to metastatic tumors, as analyzed in two published datasets (Fig. S1B) [8, 9]. Along these lines, we could confirm the tumor-specific hypermethylation of all seven markers with high significance in the publicly available cancer genome atlas PCa dataset TCGA-PRAD (Fig. S1C). In addition, we inferred 85 methylation markers from the TCGA-PRAD methylation data including PCa (*n*=498) and adjacent normal tissue (*n*=54). We selected suitable regions for marker evaluation containing at least three neighboring CpG sites, which were significantly differentially methylated (*p* < 0.05), showed low methylation in the controls (below 20%) and a methylation difference of at least 15% between controls and tumor. Out of the total 92 candidate regions, 80 were located in promoter regions (-1500bp upstream to +1000bp downstream of the transcription start site) of the respective genes, with 65 out of the 80 located in CpG islands. The remaining 12 candidate regions were located in intergenic regions or gene bodies (Table S2).

### Identification of a methylation classifier for metastatic castration resistant prostate cancer in liquid biopsies of patients

To test the 92 identified methylation markers for their suitability to detect PCa-specific DNA methylation of ctDNA in plasma samples of patients, we used a high-throughput methylation sensitive restriction enzyme (MSRE) assay [10]. This method is based on the selective digestion of unmethylated DNA, and can be applied in a multiplex setting detecting DNA amounts as low as 10 copies and 0.1-1% of methylated DNA in an unmethylated background.

First, we analyzed a total of 174 plasma samples, including patients with benign conditions (*n*=48), localized PCa (*n*=65) and mCRPC (*n*=61) (Table S1). One sample from the benign cohort was removed from further analysis due to poor performance in the MSRE-qPCR. Relative methylation values were calculated based on percentage of methylation ratios (PMR), where samples were normalized for input DNA and two control assays indicating 100% methylation. Unsupervised clustering revealed high levels of methylation in most mCRPC plasma samples, whereas normal and localized PCa plasma samples showed very low methylation values and were clustering together with the PBMC control (Fig. 1B).

Next, the 92 analyzed marker candidates were used for prediction model calculations by inputting PMR values for class prediction models based on different algorithms including Diagonal Linear Discriminant Analysis (DLDA), Nearest Centroid Predictor, k-Nearest-Neighbor Predictor, Support Vector Machines and (Bayesian) Compound Covariate Predictor (BCCP/CCP). We used a cutoff *p* value of *p* ≤ 0.01 and 10-fold cross-validation. When comparing the benign with the mCRPC sample group, 83 out of the 92 markers were calculated to accurately classify between 93 and 96% of the samples to the correct group, depending on the used prediction model (Table S3). Areas under the curve (AUC) also depended on the used model and showed values of 0.968 (CCP), 0.972 (DLDA) and 0.966 (BCCP) (Fig. S2A). Similarly, comparing the localized PCa to mCRPC cohort, 83 out of the tested 92 marker candidates classified between 87 and 96% of the samples to the correct group with AUCs of 0.956 (CCP), 0.958 (DLDA) and 0.949 (BCCP) (Fig. S2B and Table S4). While no accurate prediction model could be inferred for benign versus localized PCa in general, comparisons between benign samples and PCa with a Gleason Score of 9 or above, resulted in AUCs ranging from 0.828 (CCP), 0.794 (DLDA) and 0.8 (BCCP) with 79 to 89% correct classification for 12 of the 92 analyzed marker genes (Fig. S2C, D and Table S5). Although we also observed a significant increase of cfDNA in mCRPC samples compared to benign and localized PCa samples, no correlation of DNA methylation and overall cfDNA concentration was detectable (Fig. S2E, F). Furthermore, DNA methylation outperformed overall cfDNA concentration measures in ROC analyses (Fig. S2G).

To identify a minimal set of markers, which allow for an accurate detection of mCRPC, we performed signature classifier calculations using recursive feature elimination. This resulted in a set of three marker candidates including *CHST11*, *CUGBP2* and *PCDHGC4* that accurately differentiated mCRPC from the combined group of localized PCa and benign patients (Fig. 1C). Depending on the prediction model used, this gene signature classified between 92% and 95% of the samples to the correct group with AUCs of 0.963 (CCP), 0.978 (DLDA) and 0.953 (BCCP) (Fig. 1D, Table S6). Individual genes showed AUCs of 0.982 (*CHST11*), 0.632 (*CUGBP2*) and 0.906 (*PCDHGC4*) (Fig. S2H).

Together, these data suggest that mCRPC can be identified based on methylation signatures with high accuracy, whereas organ-confined PCa with Gleason scores lower than 9 cannot be differentiated from benign samples, most likely due to limited amounts of ctDNA, which was also described in other studies analyzing DNA methylation in localized PCa patients using digital droplet PCR [11, 12]. Generally, ctDNA shows smaller fragment sizes as compared to cfDNA shed from normal cells [13, 14]. When performing fragment analysis of a subset of benign, localized PCa and mCRPC plasma samples (n=20 per group), we observed a significant shift of the mean cfDNA fragment size from 175 bp in benign and localized PCa (range 168 - 183bp) to 168 bp in mCRPC (range 145 – 179 bp) samples (Figure S2I).

Thus, our markers might be suitable to identify high risk patients, who have already developed micrometastases, which cannot be detected by regular computed tomography (CT). Interestingly, hypermethylation of *CHST11* has been found in breast cancer cell lines originating from luminal cells, whereas basal-like breast cancer cell lines showed rather hypomethylation [15]. This might also apply to PCa, which most frequently originates from luminal cells.

### DNA methylation markers differentiate treatment responsive from non-responsive patients

Therapy options for mCRPC are diverse and evaluation of response to different treatments is thus essential for treatment decision making [4, 5]. To evaluate the potential of our identified marker genes in monitoring treatment response in mCRPC patients, we performed MSRE-qPCR analyses in liquid biopsies from mCRPC patients responsive (*n*=17) or non-responsive (*n*=12) to different therapies. Therapy response was based on increasing/decreasing PSA blood levels after therapy. Liquid biopsies were taken before treatment start and following therapy (Table S7).

We detected a trend towards higher methylation in non-responder patients before as well as after treatment, whereas responder patients showed reduced methylation after treatment compared to pre-treatment samples (Fig. 1E)

Using recursive feature elimination, signature classifier prediction models calculated a gene signature of three genes (*AKR1B1*, *LDAH*, *KLF8*) to distinguish responders from non-responders following treatment. Dependent on the prediction algorithm used, this signature correctly classified 83 – 90% of patients with AUCs of 0.902 (CCP), 0.892 (DLDA) and 0.863(BCCP) (Fig. 1F, G and Table S8). Individual genes resulted in AUCs of 0.931 (*AKR1B1*), 0.765 (*KLF8*) and 0.980 (*LDAH*) (Fig. S2J). No significant difference of overall cfDNA concentrations was evident for the different patient groups before and after treatment (Fig. S2K).

When comparing post-treatment plasma samples of responders and non-responders to abiraterone acetate treatment only, we were able to correctly classify 84% of the patients based on 13 marker genes (Table S9).

In summary, we defined a methylation classifier for measuring treatment response to different therapeutic regimens based on ctDNA methylation of 3 distinct marker genes. Therapy-response monitoring is an important task for the clinical management of mCRPC. With a continuously growing number of available therapeutic options for mCRPC patients [16] it is of high importance to choose proper treatments and adjust in a timely manner upon failed response. Thus, we suggest that our identified methylation markers could allow for early detection of non-responders and indicate patient prognosis, which could allow for patient stratification to adjust treatment and prepare appropriate countermeasures.

### Prognostic potential of DNA methylation markers for progression-free survival

To test whether our methylation markers could predict clinical outcomes such as radiographic progression-free survival (rPFS), we performed survival statistics using methylation levels of our candidate genes in plasma post treatment. Kaplan-Meier survival analysis revealed significant associations of methylation and decreased rPFS for several marker candidates including the three signature genes (*AKR1B1, KLF8, LDAH*) (Fig. 1H and S3).

Next, we performed univariate and covariate cox regression analyses to determine the prognostic value of our markers for overall survival (OS) and rPFS. For OS, methylation of *CRABP2* and *TNFAIP8* were significant prognostics factors (HR 4.013 and 0.036; *p* values 0.0395 and 0.0191) in plasma of patients following treatment in univariate analysis, but did not remain significant in co-variate models including treatment or PSA levels as variables (Table 1).

**Table 1:**
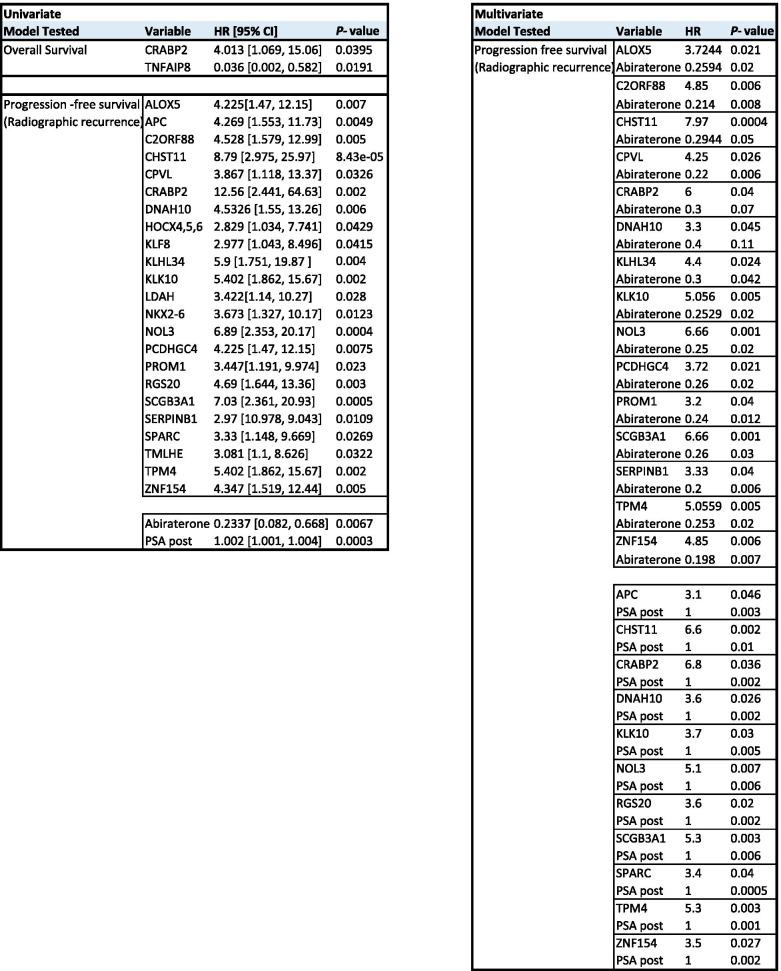
Univariate and multivariate cox regression analysis based on methylation of individual genes in plasma post treatment

For rPFS, methylation of 23 individual marker genes was a negative prognostic factor for disease recurrence in univariate cox regression analysis, with hazard ratios (HR) ranging from 2.829 (*HOCX4,5,6*, *p* = 0.04) to 12.56 (*CRABP2*, *p* = 0.002).

Since most of the responder patients underwent abiraterone acetate treatment, covariate cox regression was performed. Despite abiraterone acetate treatment showing significant predictive value for radiographic recurrence (HR 0.2337, *p* = 0.0067), methylation of 15 marker candidates remained an independent significant predictor with HRs ranging from 3.2 (*PROM1*, *p* = 0.04) to 7.968 (*CHST11*, *p* = 0.0004) (Table 1). Notably, 11 of the marker genes also remained significant in co-variate analysis adjusting for PSA as a variable (HR 3.1-6.8) suggesting that our markers are suitable for prognosis independent of PSA levels. This finding might have important implications for detecting tumors that progress regardless of PSA increase.

Taken together our findings reveal the potential of DNA methylation-based marker genes to monitor treatment response in mCRPC patients at an early stage following therapy administration. Importantly, using radiographic recurrence as an endpoint, we reveal several methylation markers that could predict tumor progression following treatment. Due to the small sample size and heterogenous treatment history of our study population, it will be important to validate our findings in an independent homogenous cohort in future studies.

## Conclusions

To this date, mCRPC remains an incurable disease. A variety of therapeutic approaches for the clinical management of mCRPC have been developed recently, and it is essential to closely monitor therapy response in those patients to maximize their survival and quality of life. Our study presents several DNA methylation-based biomarkers with the potential to detect metastatic disease and to monitor treatment response and predict disease progression in liquid biopsies of patients with advanced cancer. Aside from their usability as cancer specific biomarkers, some of our candidates might also have important biological functions for prostate cancer [17–24]. As a next step, it will be important to test the performance of our methylation markers in prospective clinical trials including mCRPC patients undergoing different treatment regimes. We envision the development of a multiplex MSRE-qPCR kit including our 3-gene signatures for routine testing of therapy response of patients with advanced PCa in clinical labs in the future.

## Supplementary Information

**Additional file 1: Table S1.** Patient cohorts and characteristics. **Table S2.** List of biomarker assays for MSRE analysis of liquid biopsies. **Table S3.** Classification results of benign versus mCRPC. **Table S4.** Classification results of primary PCa versus mCRPC. **Table S5.** Classification results of benign versus primary PCa GSc 9+. **Table S6.** Classification results of benign+primary PCa versus mCRPC. **Table S7.** Clinical data of mCRPC responder and non-responder patients. **TableS8.** Classification results of mCRPC non-responder post-treatment vs. mCRPC responder post-treatment. **TableS9**. Classification results of mCRPC non-responder post-treatment vs. mCRPC responder post-treatment PMR-values from patients that underwent Abiraterone-acetate treatment. **Table S10.** Primers used for ms-qPCR assays

**Additional file 2: Figure S1.** Validation of maker candidates. **Figure S2.** ROC analysis and cfDNA concentration. **Figure S3.** Survival analysis of responder and non-responder patients.

**Additional file 3:** Materials and Methods.

## Abbreviations

PCa: Prostate cancer
PSA: Prostate specific antigen
BPH: Benign prostatic hyperplasia
ADT: Androgen deprivation therapy
CRPC: Castration resistant prostate cancer
mCRPC: Metastatic castration resistant prostate cancer
ctDNA: Cell-free circulating tumor DNA
CTCs: Circulating tumor cells
FFPE: Formalin fixed paraffin embedded
PSMA: Prostate specific membrane antigen
PMR: Percentage methylated reference
ccfDNA: Circulating cell-free DNA
Tm: Melting temperature
MSRE-qPCR: Methylation sensitive restriction enzyme – quantitative PCR
ms-qPCR: Methylation-specific quantitative PCR
PBMC: Peripheral blood mononuclear cells
sQ: Sample quantity
DLDA: Diagonal linear discriminant analysis
CCP: Compound covariate predictor
BCCP: Bayesian compound covariate predictor
rPFS: Radiographic progression-free survival
AUC: Area under the curve
ROC: Receiver operating characteristics
CT: Computed tomography
PET: Positron emission tomography
