# Supplementary material for: Identification of tumor tissue-derived DNA methylation biomarkers for the detection and therapy response evaluation of metastatic castration resistant prostate cancer in liquid biopsies
Source: Mol Cancer. 2022 Jan 3;21:7. doi: 10.1186/s12943-021-01445-0 (PMC8722310; doi:10.1186/s12943-021-01445-0)
Supplement: Supplementary file 2 — Additional file 2: Figure S1. Validation of maker candidates. Figure S2. ROC analysis and cfDNA concentration. Figure S3. Survival analysis of responder and non-responder patients. [file 12943_2021_1445_MOESM2_ESM.pdf]

Figure S1

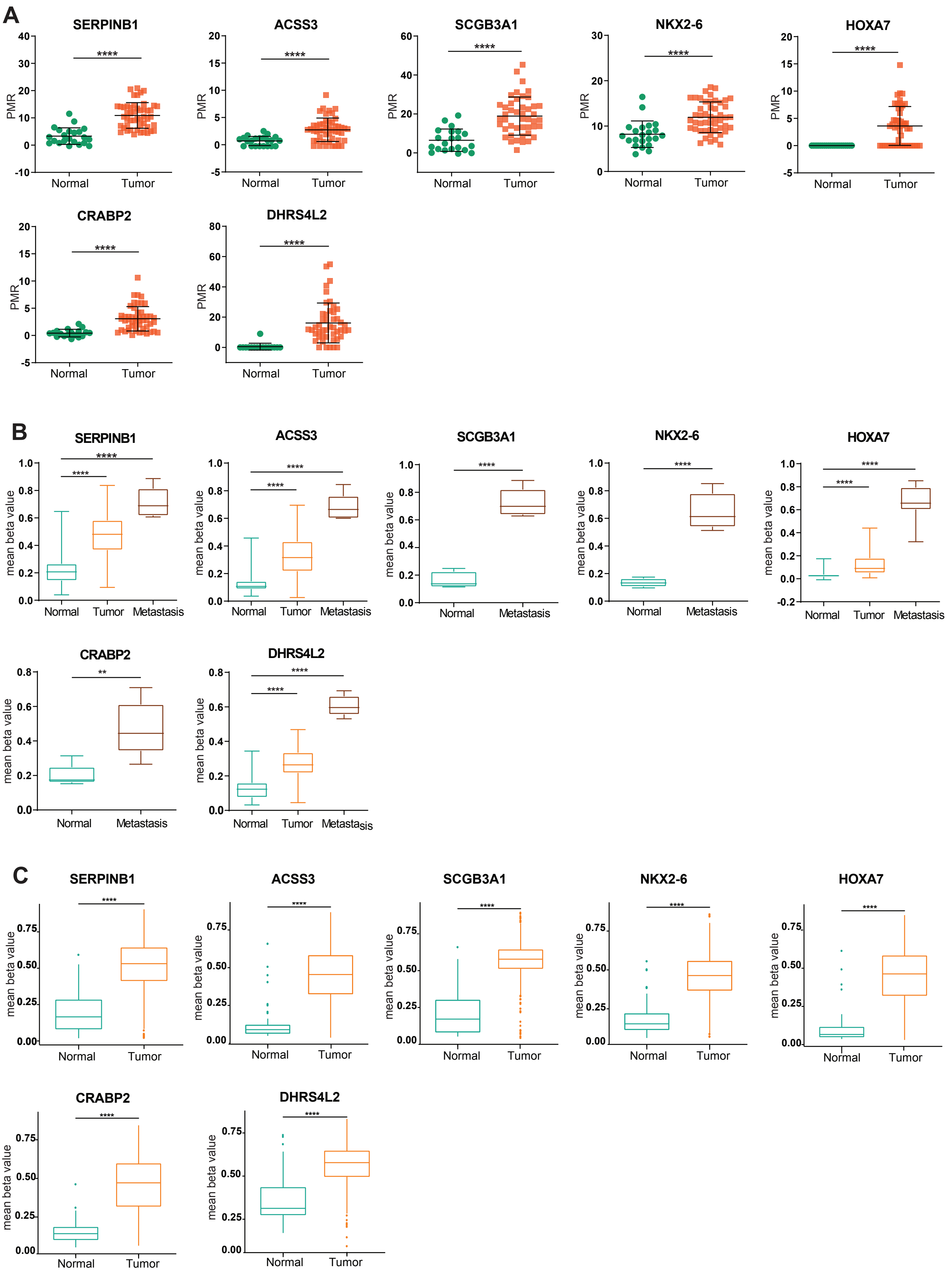

Figure S1: Validation of marker candidates. (A) Validation of differentially methylated regions in normal adjacent (n=22, green) and tumor (n=49, orange) samples by methylation-specific quantitative PCR (ms-qPCR) for seven candidate genes. Data are represented as percentage of methylated reference (PMR) values with **** p < 0.0001, based on Students t-test. (B) Validation of seven candidate marker genes in two published datasets. DNA methylation is displayed in normal (green), localized tumor (orange) and metastatic (brown) prostate cancer tissue. Differences between the groups were assessed using Students t-test and one-way ANOVA (SERPINB1, ACSS3, HOXA7, DHRS4L2: n=90 normal, n=95 tumor, n=8 metastasis, **** p < 0.0001; SCGB3A1, NKX2-6, CRABP2: n=4 normal, n=8 metastasis, ** p < 0.01, **** p < 0.0001). Please note that analyses comparing normal and localized tumors were done on Infinium® HumanMethylation 27K Microarrays, which do not cover SCGB3A1, NKX2-6 and CRABP2 genes. Normal/metastasis comparisons were performed on Infinium® HumanMethylation 450K Microarrays, covering all candidate genes. (C) The seven candidate genes were further validated in publicly available DNA methylation data from the cancer genome atlas (TCGA). The box plots show the mean methylation values of candidate genes, which were significantly (hyper-/hypo) methylated between normal and localized tumor samples (n=50 normal, n=498 tumor).

Figure S2

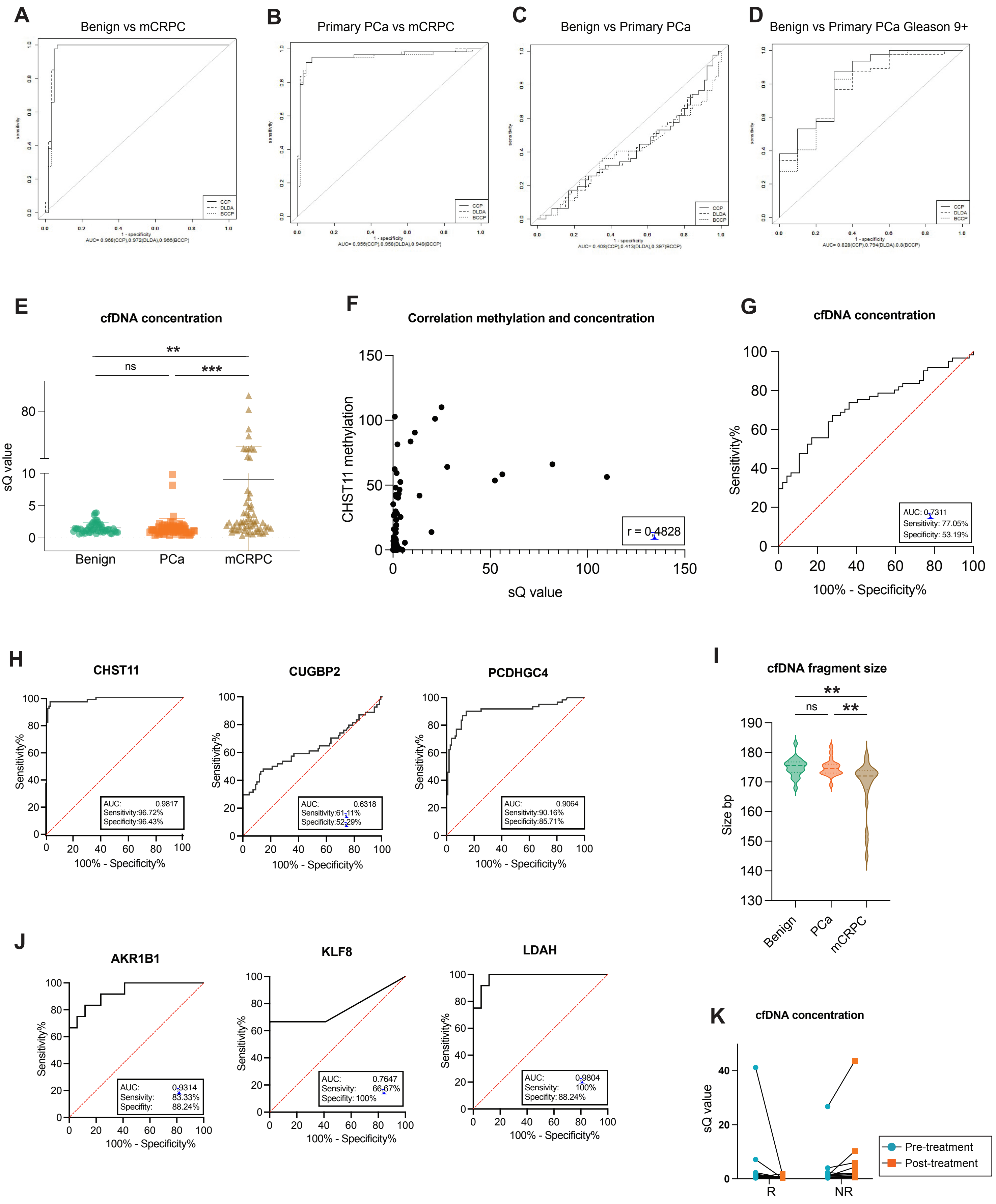

Figure S2: ROC analysis and cfDNA concentration. ROC analysis based on different prediction algorithms ((Bayesian) compound covariate predictor (BCCP, CCP) and Diagonal Linear Discriminant Analysis (DLDA)) for benign versus mCRPC (A), localized PCa versus mCRPC (B), benign versus localized PCa (C) and benign versus localized PCa with Gleason Scores greater than 9 (D), (n=47 benign, n=65 localized PCa, n=61 mCRPC, n=10 localized PCa Gleason 9+). (E) Concentration of cfDNA in benign, localized PCa and mCRPC plasma samples determined by qPCR (sQ, sample quantities calculated from DNA standard curves). (F) Exemplary correlation of DNA methylation for the CHST11 gene and cfDNA concentration. (G) ROC analysis of cfDNA concentration in plasma of benign versus mCRPC samples. (H) ROC analysis based on comparison of benign and localized PCa cohorts combined, to mCRPC patient samples for the individual genes of the calculated 3-gene signature (CHST11, PCDHGC4 n=112 benign + localized PCa, n=61 mCRPC, CUGBP2 n= 109 benign + localized PCa, n=54 mCRPC). (I) Fragment size distribution of cfDNA isolated from benign, localized PCa and mCRPC (n=20 per group; ** p < 0.01, one-way ANOVA). (J) ROC analysis based on comparisons of responder and non-responder post-treatment PMR values for the individual genes of the calculated 3-gene signature (AKR1B1, KLF8, LDAH) (AKR1B1 n=15 responder, n=10 non-responder; KLF8, LDAH n=17 responder, n=12 non-responder). (K) Concentration of cfDNA for individual responders and non-responders pre- and post-treatment (n=17 responder, n=12 non-responder , results are non-significant as determined by two-way ANOVA).

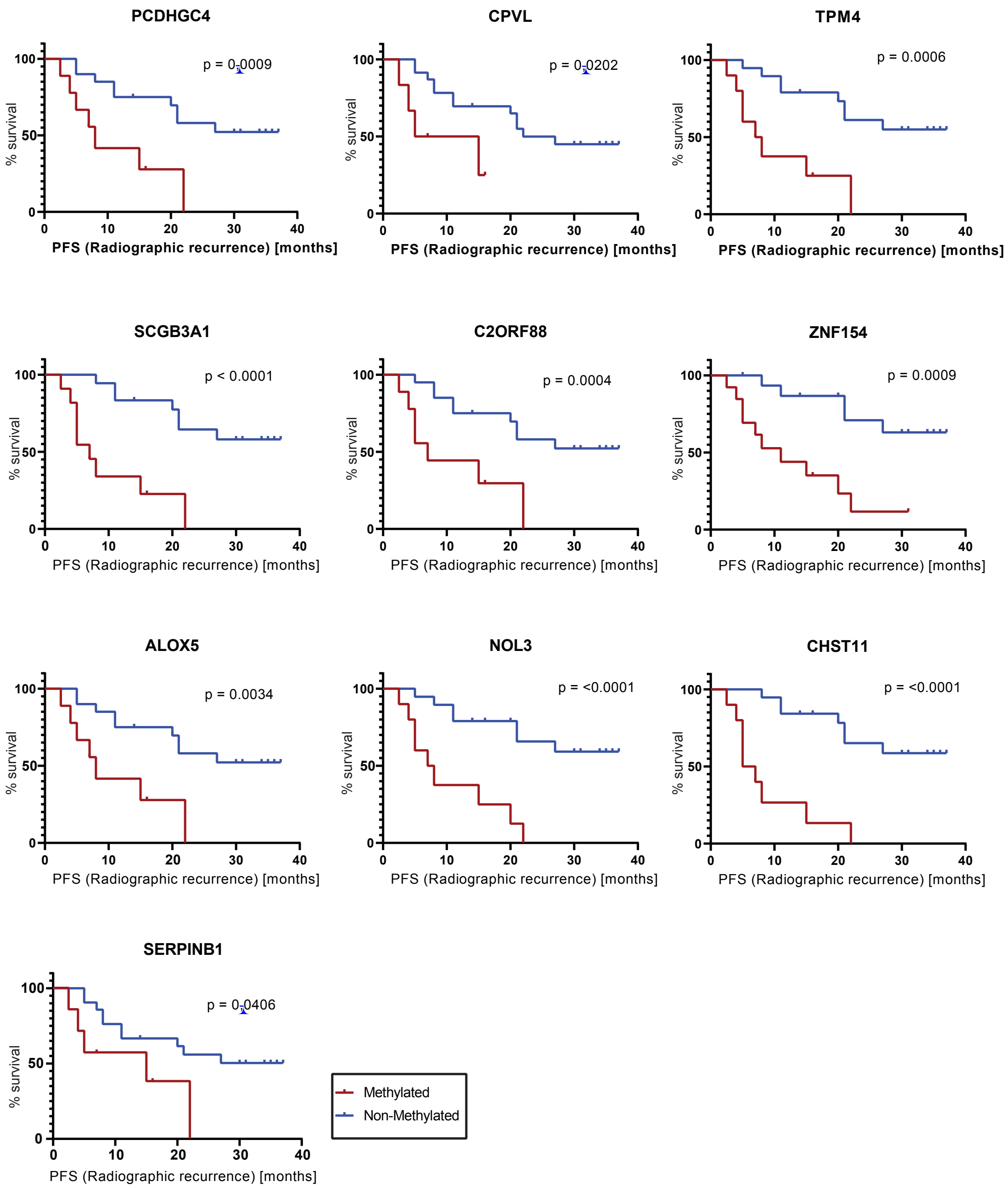

Figure S3: Survival analysis of responder and non-responder patients. Kaplan-Meier-Analysis using rPFS as endpoint based on differences in DNA methylation in post-treatment samples (n=17 responder, n=12 non-responder, p values shown on each plot calculated with Mantel-Cox-test, censored subjects indicated on plots by strokes).
