# Supplementary material for: Identification of tumor tissue-derived DNA methylation biomarkers for the detection and therapy response evaluation of metastatic castration resistant prostate cancer in liquid biopsies
Source: Mol Cancer. 2022 Jan 3;21:7. doi: 10.1186/s12943-021-01445-0 (PMC8722310; doi:10.1186/s12943-021-01445-0)
Supplement: Supplementary file 3 — Additional file 3: Materials and Methods. [file 12943_2021_1445_MOESM3_ESM.docx]

**Methods**

**Patient samples**

The initial DNA methylation analysis performed with the Infinium® HumanMethylation450K BeadChip included fresh frozen specimen from a total of nine different patients. We used six tumor and six adjacent normal prostate tissue samples isolated from matched (n=3) or non-matched (n=3+3) patients (Table S1). For validation of selected marker genes, archived formalin fixed paraffin embedded (FFPE) samples from 58 patients with clinically localized PCa (pT3) that underwent radical prostatectomy (RPE) between 1993 and 2003 at the General Hospital of Vienna were used. From those, 49 tumor and 22 normal adjacent samples were used for confirmation of our candidate markers (Table S1).

For the candidate methylation marker testing of plasma ctDNA, we included a total of 65 patients with localized PCa confirmed by needle biopsy, undergoing radical prostatectomy the day after blood draw. The benign cohort consisted of plasma from 48 age-matched men with benign conditions including benign prostatic hyperplasia. The mCRPC cohort included plasma from a total of 76 patients with confirmed metastatic disease (Table S1). Inclusion criteria for the treatment response assays were a decrease in blood PSA values of at least 65% after treatment for the responder cohort and elevated PSA levels by at least 15% for the non-responder cohort compared to pre-treatment concentrations, respectively (Table S7). For docetaxel, cabazitaxel and ^177^Lutetium (^177^Lu) Prostate Specific Membrane Antigen (PSMA) radioligand therapy treated patients, inclusion criteria included post-treatment blood draw at least 1 week following treatment end. For patients treated with abiraterone acetate, post-treatment blood draw was done at least 4 weeks after treatment start. Three patients receiving chemotherapy treatment, had their pre-treatment blood draw 1 and 3 weeks after the first chemotherapy cycle but were still included because of rising PSA values and to improve statistical power. A summary of the workflow can be seen in Fig. 1A.

**DNA isolation**

DNA from FFPE tissues was isolated using the EZ1 DNA Tissue Kit (Qiagen, Cat.Nr. 953034) and the EZ1 Advanced XL System (Qiagen, Cat.Nr. 9001874). DNA concentrations were measured using a Nanodrop 2000 spectrophotometer. Genomic DNA from cell lines was isolated using QIAamp DNA Mini Kit (Qiagen, Cat.Nr. 51304). For the isolation of circulating cell-free DNA (ccfDNA) from liquid biopsies, between 1-4ml of frozen plasma from patients with localized PCa, mCRPC and benign conditions were used, following instructions of the QIAamp® MinElute ccfDNA Kit (Qiagen, Cat.Nr. 55204). DNA was eluted in 18 µl of ddH_2_O. Isolated ccfDNA samples were immediately transferred to -80°C freezers and stored until further use. For PBMC DNA isolation, first SepMate^TM^ -50 (IVD) tubes (StemcellTechnologies, Cat.Nr. 85450) were used to isolate mononuclear cells from healthy male subjects following the user manual. DNA was then isolated using the DNeasy blood & tissue kit (Qiagen, Cat.Nr. 69504) following user manual instructions.

**Methylation-specific quantitative PCR (ms-qPCR)**

500 ng of DNA isolated from cell lines or archived FFPE tissues was used for bisulfite conversion using the EZ DNA Methylation Kit (Zymo Research, Cat.Nr. D5001). Human Methylated & Non-Methylated (WGA) DNA Set (Zymo Research, Cat.Nr. D5013) was used as methylated and unmethylated control DNA. Bisulfite-converted DNA was eluted in 100µl nuclease free ddH_2_O and used for ms-qPCR, following the MethyLight protocol [1], however, using SYBR green-based quantification. KAPA SYBR® FAST qPCR kits (KAPA Biosystems, Cat.Nr. 07959389001) with 10 ng bisulfite-converted DNA were used per reaction. For each target, one methylation specific primer pair was designed using MethPrimer software [2]. ALU-C4 methylation specific primers (methylation independent reaction) were used as input control and for calculation of percentage of methylated reference (PMR) as previously described [3]. All primers used for ms-qPCR are listed in Table S10.

**Methylation Sensitive Restriction Enzyme – quantitative PCR (MSRE-qPCR)**

Primers for MSRE-qPCR target regions were designed as previously described using Primer3 software [4]. Primers used are listed in table S2.

The MSRE-digest of ccfDNA samples was performed as previously described [5]. In brief, 75% of each sample was used for the restriction digest and the remaining 25% was used to normalize for total DNA input. Each digest was performed using 2 units of each enzyme AciI (NEB, Cat.Nr. R0551S), HpaII (ThermoFisher Scientific, Cat.Nr. ER0511), HpyCH4IV (NEB, Cat.Nr. R0619S) and Hin6I (ThermoFisher Scientific, Cat.Nr. ER0481) mixed with ultrapure water and 10x Tango Buffer (Thermo Fisher Scientific). Digest and mock digests were incubated at 37°C for 16 hours followed by enzyme inactivation at 65°C for 20 minutes. In addition to ccfDNA from patient plasma samples, genomic DNA from isolated peripheral blood mononuclear cells (PBMCs) from five male, healthy donors was isolated and used as unmethylated negative controls.

The two final steps following the digest including preamplification and qPCR on 96 x 96 dynamic arrays were performed as previously described [5]. In brief, each DNA sample, digested and mock-digested, was pre-amplified in a multiplex PCR reaction using 50nM of pooled primer pairs. The final amplification on 96 x 96 dynamic arrays was performed by running the loaded microfluidic qPCR array on a Biomark™ System (Fluidigm) using a thermal mixing step with 2 minutes at 50°C, 30 minutes at 70°C and 10 minutes at 25°C, followed by heat activation at 95°C for 15 minutes. The cycling program included 45 cycles and an annealing temperature of 65°C followed by melting curve analysis.

Similar to ms-qPCR, PMR-values were calculated for each sample. The sample quantities (SQ = amount of DNA in each reaction in ng) were calculated for each assay using standard curves. Two regions in the genes *IRF4* and *JUB* without any cutting sites for the restriction enzymes were used as 100% methylated controls.

$$PMR=100 x \frac{\frac{SQ (gene) digested sample}{SQ (gene) undigested sample}}{\frac{SQ (geomean methylated control genes) digested sample}{SQ (geomean methylated control genes) undigested sample}}$$

**cfDNA Quantification**

The concentration of cfDNA was determined by qPCR analysis using replicate standard curves of genomic DNA (20ng/run diluted in 4-fold dilution series down to 0,004882813ng/run). In addition, cfDNA fragment size and concentrations were determined with a 5300 Fragment Analyzer (Agilent) using the DNF-477 High Sensitivity Small Fragment Kit (Agilent).

**Bioinformatics analyses for DNA methylation**

DNA methylation analyses were done using the Infinium^®^ HumanMethylation450 BeadChip. Raw data were pre-processed using the minfi R/Bioconductor package [6]. Low-quality probes were removed based on a detection *p* value threshold of 0.05. In addition, probes containing SNPs or single nucleotide extensions were removed based on minfi package recommendations. Subsequently, methylation values were normalized using a combination of noob and quantile normalization methods, as implemented in the minfi package. Significantly differentially methylated CpG sites (*p* < 0.05, mean methylation difference >15%) were identified using the Limma Bioconductor package [7]. The individual CpGs were annotated to known genes using Annotatr package [8].

**Statistics**

Statistical methods used for analysis of MSRE-qPCR data were previously described [9]. In brief, BRB Array tools Version 4.6.1 (https://brb.nci.nih.gov/BRB-ArrayTools/) and R statistical software Version 3.5.1 (https://cran.r-project.org/bin/windows/base/old/3.5.1/) were used to analyze MSRE-qPCR data. We inputted PMR values for class prediction models based on different algorithms including Diagonal Linear Discriminant Analysis (DLDA), nearest centroid method, k-nearest-neighbor classification, support vector machines and (Bayesian) compound covariate predictor (BCCP/CCP). We used a cutoff *p* value of *p* ≤ 0.01 and 10-fold cross-validation. Gene signatures were calculated using minimal recursive feature elimination of the BRB Array tools plug-in and leave-one-out validation.

The unsupervised hierarchical clustering displaying sample to sample Euclidian distances based on PMR-values obtained from MSRE-qPCR with plasma samples of benign, localized PCa, mCRPC patients and PBMC DNA from healthy donors was generated by R Bioconductor ComplexHeatmap package [10].

Survival analyses based on clinical data of patients (Table S7) was performed using Kaplan Meier Statistics (GraphPad prism vs 8) and Cox proportional hazards regression. The regression analysis was performed in R 4.0.2 (https://cran.r-project.org/bin/windows/base/old/4.0.2/) using the survival package [11]. Samples with a PMR value higher than the average PMR of the PBMC control samples + 5% were defined as methylated. Samples with a PMR lower than this cut-off were defined as unmethylated. The effect of gene methylation (based on PMR values) and treatment on progression-free survival (PSA increase or radiographic recurrence) as well as overall survival (OS) was investigated. Events resulting in patient’s death or the end of the study were considered as right censored.

**Additional References**

1. Eads CA, Danenberg KD, Kawakami K, Saltz LB, Blake C, Shibata D, Danenberg PV, Laird PW: **MethyLight: a high-throughput assay to measure DNA methylation.** *Nucleic Acids Res* 2000, **28:**E32.

2. Li LC, Dahiya R: **MethPrimer: designing primers for methylation PCRs.** *Bioinformatics* 2002, **18:**1427-1431.

3. Weisenberger DJ, Campan M, Long TI, Kim M, Woods C, Fiala E, Ehrlich M, Laird PW: **Analysis of repetitive element DNA methylation by MethyLight.** *Nucleic Acids Res* 2005, **33:**6823-6836.

4. Untergasser A, Cutcutache I, Koressaar T, Ye J, Faircloth BC, Remm M, Rozen SG: **Primer3--new capabilities and interfaces.** *Nucleic Acids Res* 2012, **40:**e115.

5. Beikircher G, Pulverer W, Hofner M, Noehammer C, Weinhaeusel A: **Multiplexed and Sensitive DNA Methylation Testing Using Methylation-Sensitive Restriction Enzymes "MSRE-qPCR".** *Methods Mol Biol* 2018, **1708:**407-424.

6. Aryee MJ, Jaffe AE, Corrada-Bravo H, Ladd-Acosta C, Feinberg AP, Hansen KD, Irizarry RA: **Minfi: a flexible and comprehensive Bioconductor package for the analysis of Infinium DNA methylation microarrays.** *Bioinformatics* 2014, **30:**1363-1369.

7. Ritchie ME, Phipson B, Wu D, Hu Y, Law CW, Shi W, Smyth GK: **limma powers differential expression analyses for RNA-sequencing and microarray studies.** *Nucleic Acids Res* 2015, **43:**e47.

8. Cavalcante RG, Sartor MA: **annotatr: genomic regions in context.** *Bioinformatics* 2017, **33:**2381-2383.

9. Exner R, Pulverer W, Diem M, Spaller L, Woltering L, Schreiber M, Wolf B, Sonntagbauer M, Schroder F, Stift J, et al: **Potential of DNA methylation in rectal cancer as diagnostic and prognostic biomarkers.** *Br J Cancer* 2015, **113:**1035-1045.

10. Gu Z, Eils R, Schlesner M: **Complex heatmaps reveal patterns and correlations in multidimensional genomic data.** *Bioinformatics* 2016, **32:**2847-2849.

11. Therneau TM, Grambsch PM: *Modeling survival data : extending the Cox model.* New York: Springer; 2000.
